# Dietary Patterns Derived from UK Supermarket Transaction Data with Nutrient and Socioeconomic Profiles

**DOI:** 10.3390/nu13051481

**Published:** 2021-04-27

**Authors:** Stephen D. Clark, Becky Shute, Victoria Jenneson, Tim Rains, Mark Birkin, Michelle A. Morris

**Affiliations:** 1 Leeds Institute for Data Analytics and School of Geography, University of Leeds, Leeds LS2 9JT, UK; S.D.Clark@leeds.ac.uk (S.D.C.); fs10vl@leeds.ac.uk (V.J.); m.h.birkin@leeds.ac.uk (M.B.); 2 Sainsbury’s Supermarkets Ltd., Holborn, London EC1N 2HT, UK; Becky.Shute@sainsburys.co.uk (B.S.); Tim.Rains@sainsburys.co.uk (T.R.); 3 Leeds Institute for Data Analytics and School of Medicine, University of Leeds, Leeds LS2 9JT, UK

**Keywords:** dietary patterns, transaction data, nutrients, socioeconomic, big data, nutrition analytics, dietary assessment

## Abstract

Poor diet is a leading cause of death in the United Kingdom (UK) and around the world. Methods to collect quality dietary information at scale for population research are time consuming, expensive and biased. Novel data sources offer potential to overcome these challenges and better understand population dietary patterns. In this research we will use 12 months of supermarket sales transaction data, from 2016, for primary shoppers residing in the Yorkshire and Humber region of the UK (*n* = 299,260), to identify dietary patterns and profile these according to their nutrient composition and the sociodemographic characteristics of the consumer purchasing with these patterns. Results identified seven dietary purchase patterns that we named: Fruity; Meat alternatives; Carnivores; Hydrators; Afternoon tea; Beer and wine lovers; and Sweet tooth. On average the daily energy intake of loyalty card holders -who may buy as an individual or for a household- is less than the adult reference intake, but this varies according to dietary purchase pattern. In general loyalty card holders meet the recommended salt intake, do not purchase enough carbohydrates, and purchase too much fat and protein, but not enough fibre. The dietary purchase pattern containing the highest amount of fibre (as an indicator of healthiness) is bought by the least deprived customers and the pattern with lowest fibre by the most deprived. In conclusion, supermarket sales data offer significant potential for understanding population dietary patterns.

## 1. Introduction

Poor diet is responsible for 90,000 deaths per year in the United Kingdom (UK) alone, equating to 1 in 7 of all deaths [1]. It is a major modifiable risk factor for a range of non-communicable disease [2] and poor diet can additionally leave people more susceptible to infectious diseases [3]. There are numerous drivers of diet related behaviours including biological, economic, environmental and societal [4]. These factors can contribute to inequity with respect to access to a nutritious balanced diet. Access to food and drinks will systematically vary by country due to availability of products within local food systems, and to a different extent vary locally within countries [2,5].

Diet is measured based on consumption of food and drinks, typically self-reported through a food diary or food frequency questionnaire which capture typical diet behaviours. However, these methods present limitations around error and bias in reporting [6]. When time and money permit, these dietary assessments may be administered by a trained nutritionist or dietitian, in the form of an interview. Traditionally such food records are collected in paper form and then foods and drinks are coded by a researcher in specialist dietary assessment software. Increasingly, online tools are available for individuals to record their food and drink consumption, with the benefit of features such as typical pack sizes and portion size images, ‘commonly consumed together’ prompts and bar code scanning of products [7,8]. Rich databases lie behind these tools containing nutrient composition data and automating the coding of the diaries. Dietary surveys in the UK, such as the National Diet and Nutrition Survey (NDNS) [9], or cohort studies [10] designed to investigate diet–disease relationships are often used to inform policy decisions. These are excellent resources, even though they are likely to contain biases such as reporting bias and selection bias, and relatively modest sample sizes.

An alternative to recording consumption is to capture food and drink purchases. Here there is an assumption that food and drinks purchased are later consumed. Purchase records or sales data could provide a better reflection of household dietary patterns; however, they are unlikely to be sensitive enough to reflect individual behaviour, except where people live alone. The Family Food Unit of the Living Costs and Food Survey (LCFS) captures household expenditure from purchase records, so the principle of using purchase rather than consumption records is established [11]. Additionally, market research companies such as Kantar and Nielsen use panel surveys where participants scan their foods and drinks purchased, again offering a novel method for assessing diet using purchase records, but at a significant cost to the research team [12]. These methods reduce mis-reporting caused by forgetting what has been consumed, or inaccurate recollection of portion size; however, they do not remove social desirability bias, as participants can “choose” not to scan certain items, for example snacks and soft drinks that may be bought away from home [13]. Research that utilised purchase records from collection of till receipts to estimate nutrient composition showed promise, although did not reduce the data collection burden [14]. 

There is increasing appetite around the world to use sales data direct from supermarket loyalty card schemes or transaction records [15]. These data have been used in the evaluation of randomized control trials, with a modest number of participants and with their consent [16]. There are examples of sales data at scale from loyalty card transactions in location planning research, rather than in a nutritional context, using data to predict demand in tourist destinations [17]. We have additionally seen transaction data in sustainability research investigating purchase patterns of organic and free range foods [18]. More recently research is emerging using transaction data to better understand nutrition and related health outcomes [19,20]. Research from Finland has investigated the representativeness of data from supermarket loyalty card schemes, showing that women in their forties are overrepresented and that food expenditure recorded on the cards was equivalent to two thirds of the nationally estimated averages [21]. However, they report that these data present great potential to collect data dynamically and at scale in order to enable timely societal dietary insight to be generated.

Access to supermarket transaction data for academic research is challenging due to the commercial sensitivity of such data. In most cases this means that research is prohibited, but where partnerships and research have emerged, transaction data are aggregated in order to reduce disclosure of information which may provide a competitive advantage to other supermarkets. Alternatively, data are only provided for a particular sample of customers, a specific region or specific age group. In some cases, the retailer chooses to keep their identity secret. In spite of these challenges, the use of sales data in nutrition research offers substantial opportunity to transform our understanding of population dietary behaviour.

The aim of this paper is to use sales data from a loyalty card scheme to identify dietary purchase patterns and their associated nutrient profiles and how these vary according to sociodemographic characteristics. We hypothesise that distinct dietary purchasing patterns will be identified from the typical purchasing behaviours of different demographic groups.

## 2. Materials and Methods

### 2.1. Subjects

Participants in this research were 311,972 unique Nectar loyalty card holders that reside in the Yorkshire and Humber region of the United Kingdom. All transactions for the whole of 2016 were included in this research. Participants were identified as “primary” shoppers, where we have estimated that they complete the majority of their shop with Sainsbury’s supermarket. Two conditions were set to identify these shoppers. Firstly, they needed to have shopped with the retailer on a least 10 occasions during 2016. This rule removes those loyalty card holders who just made occasional purchases, e.g., when on vacation or at Christmas. The second condition was that they must purchase from a variety of food groups. Each item purchased is categorised into one of 15 groups. Eleven were derived from the Living Cost and Food Survey (LCFS) categories: Carbohydrate Products; Meat and Fish; Dairy; Fats; Fruit; Salad/Vegetables; Potatoes; Sweets: Other; Non-alcoholic drinks; Alcoholic drinks and a further four generated by the research team: Ready Meals; Baby Food; Cakes and Biscuits; Crisps and Nuts. Only loyalty card holders that purchased from at least seven of these 15 categories, or purchased ready meals and from three other categories, are included in the cohort. This excludes more casual shoppers, e.g., those who habitually only purchase a meal per deal (typically composed of a sandwich, a drink and a snack) or top-up a main shop made elsewhere with occasional purchases of bread, milk or snacks from the retailer. The sample of loyalty card customers is therefore designed to include only those who regularly do their main shop with the retailer.

In addition to transaction records, we received information on the gender of the loyalty card holder and their age band. We also received a neighbourhood geographic identifier, an output area (typically composed of 125 households), to facilitate linkage to area characteristics including 2015 Index of Multiple Deprivation (IMD) ranked deciles [22] and the Output Area Classification (OAC) geodemographic classification [23]. This OAC classification uses 2011 Census data from a range of domains to group similar types of characteristic within an area.

### 2.2. Transaction Records

The transaction data provided included records that linked sales to the loyalty card via a hashed ID to maintain anonymity of customers. Information on product sales provided include: the stock keeping unit (SKU) code that identifies the product; the number of these items that were purchased; the price paid for the items; and, where the item was sold by weight, its weight in g.

During our sampling time frame from the 1 January to 31 December 2016 the retailer carried just over 25,000 products, as identified by their SKU, and in total they sold over 334 million items to our sample of loyalty card holders. Following initial inspection, we identified unusual purchasing patterns at the extremes of loyalty card spending, therefore we removed these outliers, based on information from the 2016 edition of the Family Food Survey (FFS), for the Yorkshire and the Humber region [22] in order to estimate a reasonable upper bound on what a family may spend on non-alcoholic food items at a supermarket through the year. This upper bound is defined as 1.5× the inter-quartile range beyond the upper quartile (a common criteria to identify large outliers in box plots). Thus, any loyalty card holder that spent more than this amount on non-alcoholic food items with the retailer was excluded from our sample. This removes 2.04% of loyalty card holders, leaving *n* = 305,616. For consistency we additionally removed customers spending in the bottom 2.04% of spends leaving a final sample for analysis of 299,260 loyalty card holders.

### 2.3. Nutritional Information

Nutrient composition data were linked to our sales data at product level. These were “back of packet” values per 100 g or per 100 mL and included: Energy (kcal); Carbohydrates; Protein; Fats; Saturated fats; Salt; Sugar and Fibre. These nutrient composition data were obtained from the supermarket own brand databases and from the commercial brandbank database for branded products. Where products still had missing nutritional information, we sourced these from the McCance and Widdowson food tables [24], or by searching the internet, recognising that nutrient information may not always match temporally. It was not possible to link nutrient composition information for 2.1% of the 2016 sales, which were associated with the least popular products.

Nutrient composition for the sales were calculated using values per 100 g (or mL) and product weight. Product weight data was sourced from the retailer or mined from the product description in our transaction files. We used the nutrient information for foods as sold, rather than as eaten, as we could not estimate cooking methods or waste. We did not account also for edible portions of food. It was not possible to generate weight data for 0.05% of total items sold in 2016.

Each product was assigned one of the 82 detailed categories that include the LCFS sub-categories, in addition to researcher generated categories: Savoury snacks; Ready Meals; Baby Food; Meat Alternatives; Dairy Alternatives. We were unable to categorise 0.1% of items sold.

### 2.4. Analysis

#### 2.4.1. Identifying Dietary Patterns

k-means clustering was used to identify dietary patterns in the sales data. Loyalty card holders are represented by the distribution of their purchase volume (weight in grams) in the 82 categories that include food, non-alcoholic and alcoholic beverages. Provided with a number of classes to find, k-means attempts to group together loyalty card holders with similar patterns of purchasing. For k groups, the method begins with k starting points defined by a random composition amongst the 82 categories. Each loyalty card holder is then allocated to the starting point that they are most similar to, and the mean composition across the 82 categories in each of the k groups is calculated. These calculated means then replace the initial k starting points, and each loyalty card holder is re-allocated to which of these updated k points they are most similar to. This iterative process of re-allocation and calculation is repeated until no loyalty card holder changes their group membership between iterations, or a maximum number of iterations are reached. When this process has finished the k-means solution provides the typical composition for each of the k groups and the group that each loyalty card holder belongs to. 

Whilst this process is largely automatic, an important requirement is that the number of groups, k, is known. Typically, this is not the case and some judgement is required to decide on a suitable value for k. The “quality” of a solution given a value of k can be accessed via a within group sum of squares (WSS). This quantity is a measure of how similar loyalty card holders within a group are, a lower value signifying that the loyalty card holders are more similar to each other. By design, as the number of groups increases this value will not decrease, meaning that, as k increases, the quality of the solution will not deteriorate, so that looking for a measure of maximum quality (i.e., a minimum WSS) will not be possible. Instead, what is adopted in practice is to identify when this improvement in quality (reduction in WSS) as k increases becomes negligible or constant. This is best done using a scree plot, with the value of k along the x axis and the value of WSS for k on the y axis. The value of k where this plot develops an “elbow” indicates negligible improvement and is chosen as the value for k. 

Another consideration with k-means is that the method works best when the ranges of the values within the categories (e.g., the weights of different foods) are similar (to prevent unequal weight been given to some categories relative to others) and not skewed (k-means looks to form “circular” groupings rather than elongated ones). This is achieved here by range standardising each category, so that the values lie within a range from 0.0 to 1.0, and applying the inverse arc-sin square-root transformation to reduce skewness.

Once a value of k is determined then each loyalty card holder is assigned to a group and, to help identify the nature of the group, the (untransformed) mean volume purchased in each category for members of that grouping can be calculated. For example, in one group the average purchase of meat amongst its members may be much lower than the overall sample, or in another group purchase of ready meals may be higher.

#### 2.4.2. Profiling Dietary Patterns

The dietary patterns identified by the k-means will be further described, according to their nutrient composition, by the demographic characteristics of the loyalty card holders associated with each pattern and by the typical area characteristics of the loyalty card holders. The nutrient composition will provide an estimated daily consumption of the eight back-of-pack nutrients, calculated by dividing the average total nutrient for each dietary pattern by 366 (as 2016 was a leap year). Adult reference intake will be provided in the results for comparison [25]. Tests for statistical significance in difference were not carried out. Due to the large sample size, all differences are likely to be highly statistically significant. We interpret difference in terms of a clinically meaningful difference.

### 2.5. Data Sharing

Due to the commercial nature of the data used in this research, it is not possible for data to be published alongside the manuscript.

### 2.6. Ethical Approval

This research was reviewed by the University of Leeds ethics committee reference: AREA 18-050.

## 3. Results

### 3.1. The Sample

**Figure 1:**
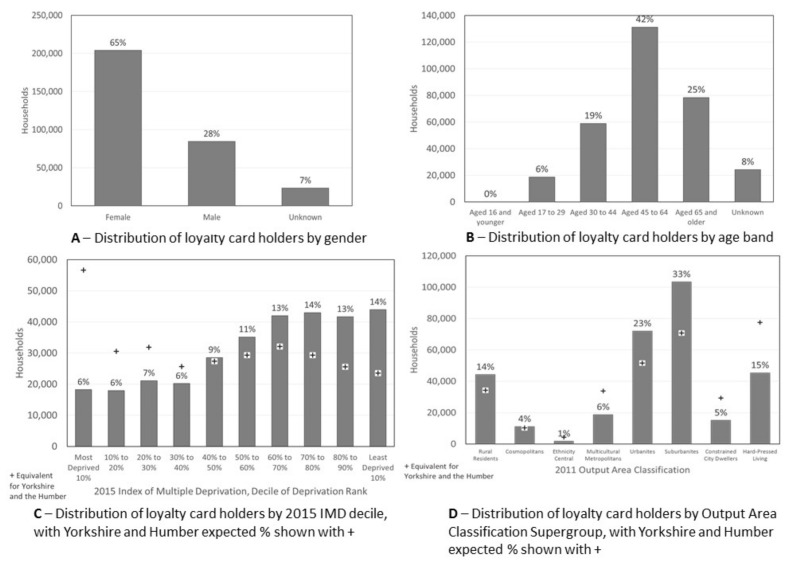
Characteristics of our loyalty card holders.

Our sample of 299,260 loyalty card holders includes large numbers in each demographic group. However, women (Figure 1A), those aged 45–64 (Figure 1B) and those living in areas within the six least deprived deciles of deprivation (Figure 1C) are over-represented. Customers typically living in areas characterized by the 2011 OAC classification as Rural residents, Urbanites and Suburbanites are also over-represented (Figure 1D). The median frequency of shopping occasions for our classification sample was 53 with interquartile range 33 to 82.

### 3.2. Dietary Patterns

**Figure 2:**
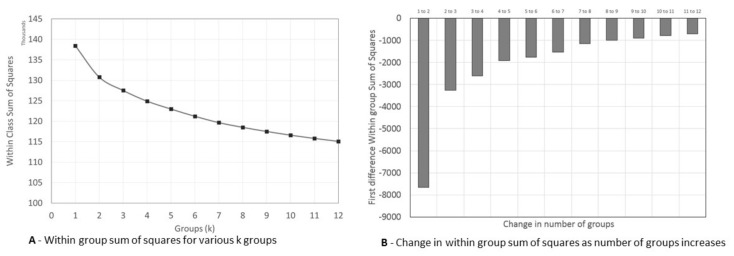
(**A**) Scree plot and (**B**) change in within group sum of square for identifying number of dietary patterns.

Using the scree plot (Figure 2A) and change in WSS (Figure 2B) we identified seven distinct dietary patterns. The scree plot looks inconclusive, but for the first differences, the reduction in WSS when moving from 6 to 7 groups is −1543, but thereafter there are much smaller step change reductions of around −1000. This provides support for having seven groupings within the data. 

**Figure 3:**
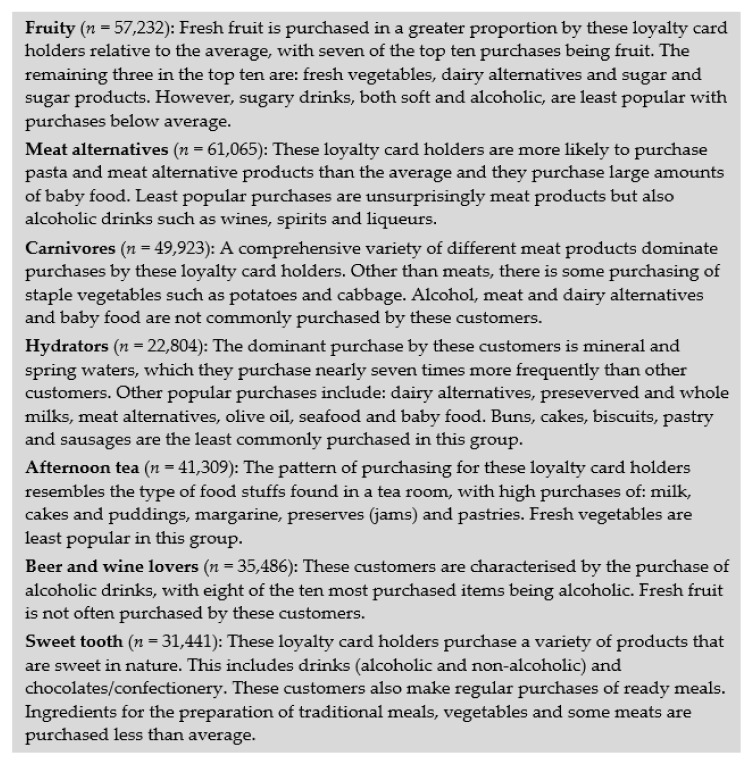
Summary of the dietary pattern contents. Please see Appendix A for more details.

Features of the seven dietary patterns are presented in Figure 3. Further information and radar plots for each pattern are provided in Appendix A.

### 3.3. Demographic Characteristics Associated with Each Dietary Patterm

**Figure 4:**
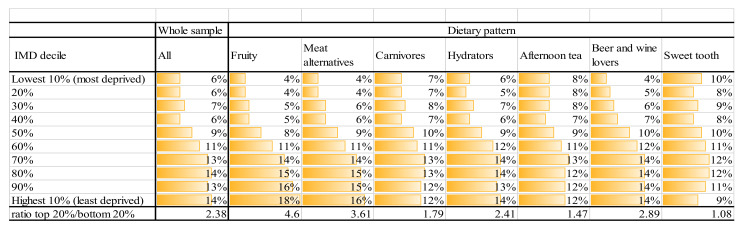
Distribution of groups of loyalty card holders amongst Index of Multiple Deprivation (IMD) deciles.

Dietary patterns are ordered by their approximate healthfulness, using amount of fibre as a proxy for healthfulness. Figure 4 shows how each dietary pattern is purchased within deciles of deprivation, with variation across deciles of deprivation. We see that the “Sweet tooth”, “Afternoon tea” and “Carnivores” patterns are over-represented in the most deprived decile and the “Fruity” cluster is over-represented in the 3 least deprived deciles. Additionally, the bottom row of Figure 4 provides the ratio of the percentage of loyalty card holders in the top (least deprived) two deciles relative to the bottom (most deprived) two, clearly highlighting where patterns are more likely to be purchased by the most deprived (lowest number) or least deprived (highest number). The “Meat alternatives” pattern is most likely to be purchased by customers living in the least deprived areas and the “Sweet tooth” in the most deprived.

**Figure 5:**
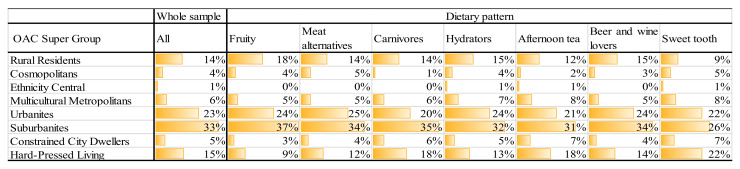
Distribution of groups of loyalty card holders amongst Output Area Classification (OAC) Super Groups.

The same type of social patterning is observed in Figure 5 where the dietary patterns are profiled against the OAC classification. The “Sweet tooth” customers are under-represented in Rural residents neighbourhoods, but overrepresented in those that are categorised as Hard-pressed living. The “Fruity” customers are over-represented in Hard-pressed neighbourhoods, which is coupled with over representations among the Rural residents and Suburbanites.

Table 1 provides a breakdown of the daily nutrient purchasing of each dietary pattern. Those within the “Carnivores” pattern purchase the highest daily amount of calories, just over the UK adult reference intake. By contrast, “Sweet tooth” customers gain the lowest amount of their daily nutritional requirement from this supermarket. None of the patterns contain the recommended daily fibre intake, but the “Fruity” pattern contains the most at 21 g/day, just over two thirds of the daily recommended amount. In contrast, the “Sweet tooth” pattern only contains 10 g/day fibre, one third of the daily recommended amount. None of the patterns contain enough carbohydrate, with the highest carbohydrate purchases in the “Meat alternatives” group, buying 80% of the recommended amount. All of the patterns, except “Sweet tooth” contain enough protein. “Afternoon tea”, “Sweet tooth” and “Beer and wine lovers” meet the target of <70 g/day fat; however, all patterns exceed the daily saturated fat recommendations, with the “Carnivores” pattern most exceeding both fat and saturated fat. While the “Sweet tooth”, pattern is the least healthy, with the lowest fibre content, and typified by sugary food and drinks, this cluster purchase on average, the least amount of calories, which appears to be the reason they do not exceed the daily fat target and have lower sugar purchases than other patterns. Conversely, “Fruity”—the healthiest pattern—contains the highest amount of sugar, likely explained by natural sugars from fruit. That said, despite being dominated by fruit purchases, it also contains above average purchases of sugar.

## 4. Discussion

We present a novel analysis of large and complex supermarket transaction data that identifies dietary patterns purchased in the UK. We believe this to be the first work of its kind using such detailed food transactions with associated nutritional information. We were able to generate data driven dietary patterns for ~300,000 loyalty card holders across one region of the UK and profile these by average daily nutrient intake. While the sample was biased towards certain demographic characteristics, data were available for large numbers of loyalty card holders in each sub-group.

### 4.1. Dietary Pattern Insight

Our sample only included shoppers that we believe do the majority of their food shopping at Sainsbury’s supermarket. We were interested in capturing an indicator of habitual intake, akin to a diet diary or food frequency questionnaire, rather than looking at occasional shoppers, or those who only buy in limited food groups. Our method for identifying these customers used both a frequency metric and an indicator of variety, based on national survey categories. Whilst we set a threshold of at least 10 shops with the retailer, in reality our cohort of loyalty carders shopped more frequently, with a median of 53 shopping occasions throughout the year. Subgroup analysis (not reported here) showed that loyalty card holders shopping at least biweekly had slightly higher daily energy purchases, at 1887 kcal/day compared to the whole cohort at 1757 kcal/day.

Our data include food and beverage purchases, including alcohol. Two of the dietary patterns: “Beer and wine lovers” and “Sweet tooth” were driven by alcohol sales, of different types. These two patterns were associated with the two lowest daily calorie intakes, suggesting perhaps that while these shoppers met the criteria for purchasing regularly from a range of different food groups, their main priority during their shop with this retailer was for alcohol purchases. Characteristics of the loyalty card holders purchasing each of these patterns were quite different, with the “Sweet tooth” pattern more commonly containing customers living in a neighbourhood characterised by the OAC geodemographic classification as Hard-pressed living. The “Beer and wine lovers” dietary pattern is distributed across OAC groups in a similar way to the total cohort of loyalty card holders, with no noticeable over or under-representation.

All but the “Carnivores” pattern contain less than adult reference intake for energy (2000 kcal), but all except the “Sweet tooth” and “Beer and wine lovers” contain more calories than the mean adult woman’s intake in 2016 reported by the NDNS of 1632 kcal [9]. It is known that the NDNS under-reports dietary intake, and results here are broadly supportive of that, with our cohort average intake of 1762 kcal/day. 

The “Fruity” pattern is interesting, with seven of the top ten purchased items being types of fruit. This pattern is most popular among Rural residents and in Suburbanite neighbourhoods, which are typically quite affluent. It contains the highest daily fibre intake, but this is still way below the daily recommended intake. Fibre does not form part of the adult reference intake guidance, nor is it mandated to appear on the back-of-pack of food products [26]. In 2015 the UK Scientific Advisory Committee on Nutrition recommended an increase in daily fibre intake to 30 g/day in the UK following a comprehensive review of the literature [27]. These sales data are from 2016, the year following the report. In the case of fibre, it would be interesting to follow up with more recent data to see how consumers have responded to these recommendations.

Conversely, in the case of salt, where recommendations to reduce salt intake have been around for much longer, only the “Carnivores” pattern exceeds the daily recommended salt intake, and this is by 1 g at an average of 7 g/day. This is suggestive that the highly publicised guidance and product reformulations—with voluntary targets introduced in 2005 and the most recent legislation brought in in 2015—are largely successful at helping consumers achieve targets [28]. Considering that loyalty card holders may be buying for more than one person, this implies that salt targets are likely to be met by all, and that fibre targets are even further from being achieved.

### 4.2. Study Strengths and Limitations

The most striking strengths of this research are the large sample size and the objectively measured sales records for dietary intake. The work presented here highlights the potential scope of sales data for identifying dietary purchase patterns. The transaction data are comprehensively matched to detailed nutrient composition data at the product level, providing a powerful dataset to investigate nutritional patterns and trends. These types of data would enable comprehensive modelling of proposed nutritional policies, such as the planned legislation expected to be announced in summer 2021 to restrict price and location promotions of high fat, sugar and salt products [29]. It has been recognised that there are limitations in the data that retailers hold which would enable them to successfully implement these new rules across their whole product portfolio [30], but this new dataset, which combines sales and nutrient composition data, would make that easier and would also enable, for the first time, quantification of the impact of legislation in objective sales weighted data, not subject to the limitations of self-reporting, and therefore unlike survey and panel data.

These linked sales and nutrient data, with demographic characteristics, additionally make a bold step towards digitising the food system. While they may only represent part of the consumer facing retail environment, this could be the starting point for combination with other sources of retail data from the food supply chain [5].

However, this is matched by some notable limitations. These data are only from one supermarket, which as the results demonstrate is used by people residing in areas across all deciles of deprivation, but over-represented in the least deprived areas. The loyalty card holders are predominantly women, which is the same observation found by Nevalainen, Erkkola [21] in Finland, suggesting that women still take on the main responsibility of providing food for their family, or that women register for cards that men also use, and they are middle aged, again consistent with the Finnish study.

In our loyalty card data, we do not know whether the loyalty card holder is shopping for themselves, or for a family. This significantly limits the conclusions that can be drawn from our results and the reason why we only present descriptive statistics. Average daily nutrient intakes were calculated as if the loyalty card holder were living alone, but we can see from the contents of the patterns that some customers are likely to be buying for a family. With this in mind, the nutrient profiles presented in Table 1 are likely to under-represent the daily intake of these consumers. Compared with the NDNS, the average daily energy intake is still >100 kcal greater per day. That said, it is widely recognised that the NDNS under-reports diet in the UK. Supermarket sales data do not capture out of home food purchases that dietary surveys have the potential to capture—yet still our study demonstrates a higher daily calorie intake than NDNS. LCFS purchase data from 2016/17 suggests that 88% of purchases, by volume (g or mL) are for home consumption, compared to 12% eating out [31].

Another limitation is that we do not know whether the loyalty card holder does all of their shopping at Sainsbury’s, nor whether they use their loyalty card each time. They may forget their card, or do “top-up” shops from another retailer. 

Data driven dietary patterns are beneficial as they identify patterns relative to the data subjects’ behaviour, rather than trying to score them against a pre-existing dietary pattern. Being data driven, using detailed food categories, they are more difficult to compare to patterns observed in other studies, generated from subjects with different consumption or purchasing behaviours. That said, there are common themes in our patterns seen in many other studies, for example: presence of a carnivore pattern, a sweet tooth/snacking pattern and a meat alternative, or vegetarian pattern [32,33]. Our patterns differ from many previous studies, due to the inclusion of alcohol in the cluster generating process, which we believe to be a strength as alcohol contributes to energy intake. The naming of dietary patterns can be subjective and as such we have included detailed information on the content of the patterns in Appendix A for transparency.

### 4.3. Future Work

We hope that this study is the first of many that make use of supermarket sales data to generate insight into food purchasing behaviours around the world. In order to translate food purchase behaviours to dietary consumption, evaluation of transaction records as a source of dietary assessment is required [34].

It is important to better understand the bias in these data, with respect to demographic characteristics of the shoppers, household composition and how much shopping is carried out at this retailer using a loyalty card. Incorporating an indication of out of home purchases to sales data would facilitate understanding of total dietary intake. 

Comparison with established dietary patterns or frameworks, such as the Eatwell Guide [35], nutrient density measure [36] or healthy eating index [37], would facilitate comparison to patterns generated in other research, for example cohort studies. 

Linkage of dietary purchase patterns to health records, with explicit consent, to better understand diet disease relationships could be paradigm shift in prevention and management of non-communicable disease. 

## 5. Conclusions

Sales data recorded by supermarket loyalty cards, linked with nutrient composition records and demographic characteristics, offer potential to understand food purchases at a scale not seen before, which unlocks the potential for more robust analysis to inform diet related policy. Further work is required to better understand the biases within these data and to extend data providers to include representation from other retailers and the out of home sector.

## Figures and Tables

**Table 1:** Daily nutritional values for loyalty card holder by group. * Fibre not included in adult reference intake values, but UK recommended intake included below.

Nutrient	Adult Reference Intake	All	Fruity	Meat Alternatives	Carnivores	Hydrators	Afternoon Tea	Beer and Wine Lovers	Sweet Tooth
Energy (kcal)	2000	1757	1818	1869	2026	1729	1632	1585	1384
Fat (g)	<70	72	75	76	89	72	66	61	56
Saturates (g)	<20	28	29	29	35	27	27	23	22
Carbohydrates (g)	At least 260	186	197	207	203	183	189	141	155
Sugars (g)	90	87	97	93	90	86	91	61	72
Salt (g)	<6	5	6	6	7	5	5	5	4
Protein (g)	50	66	69	70	83	65	58	57	47
Fibre (g)	30 *	16	21	18	18	16	14	12	10

**Figure A1:**
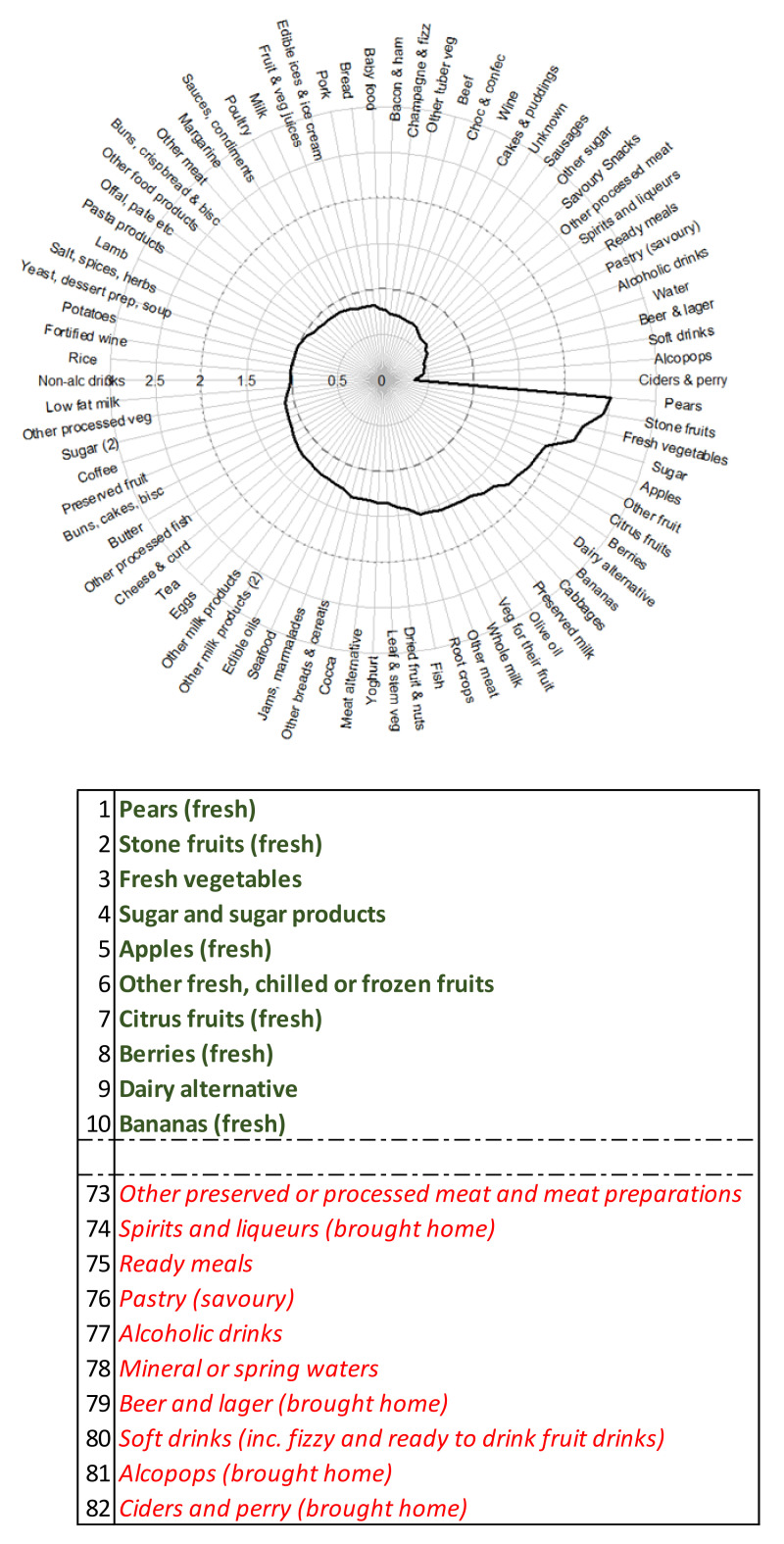
Fruity (*n* = 57,232).

**Figure A2:**
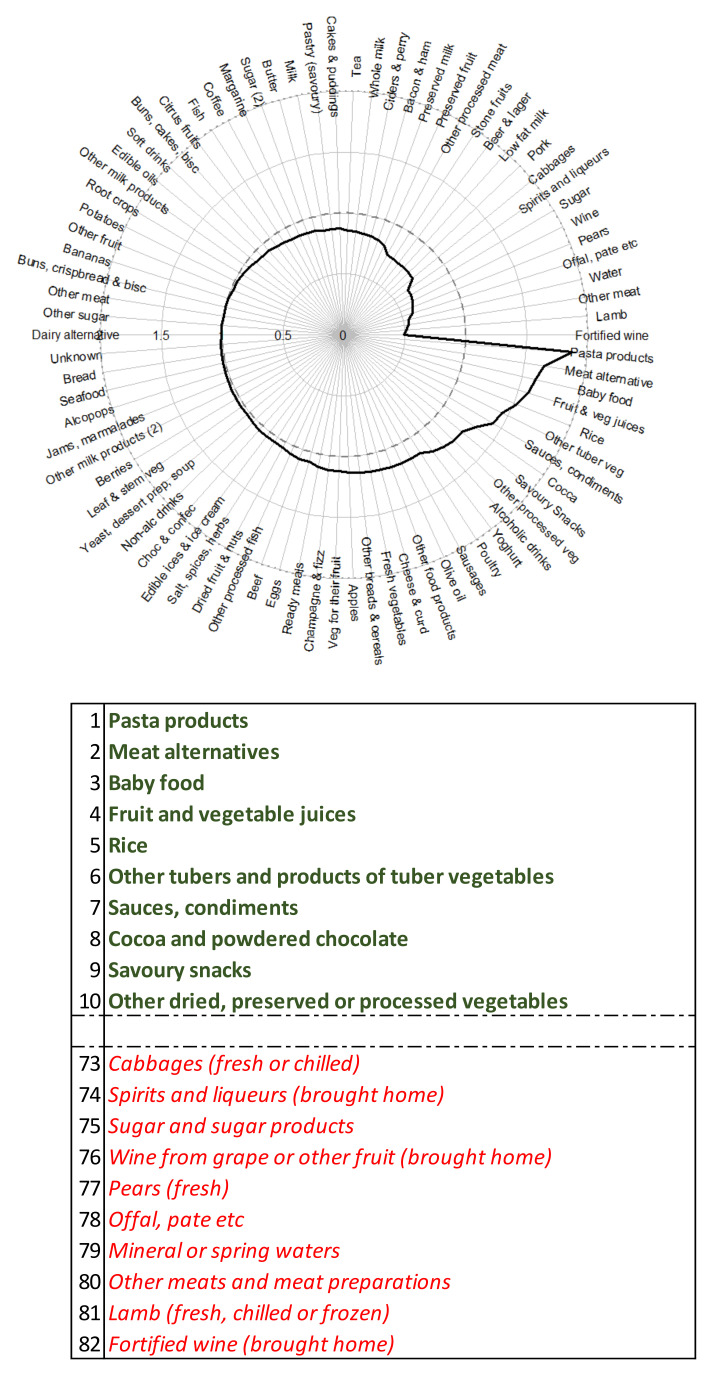
Meat Alternatives (*n* = 61,065).

**Figure A3:**
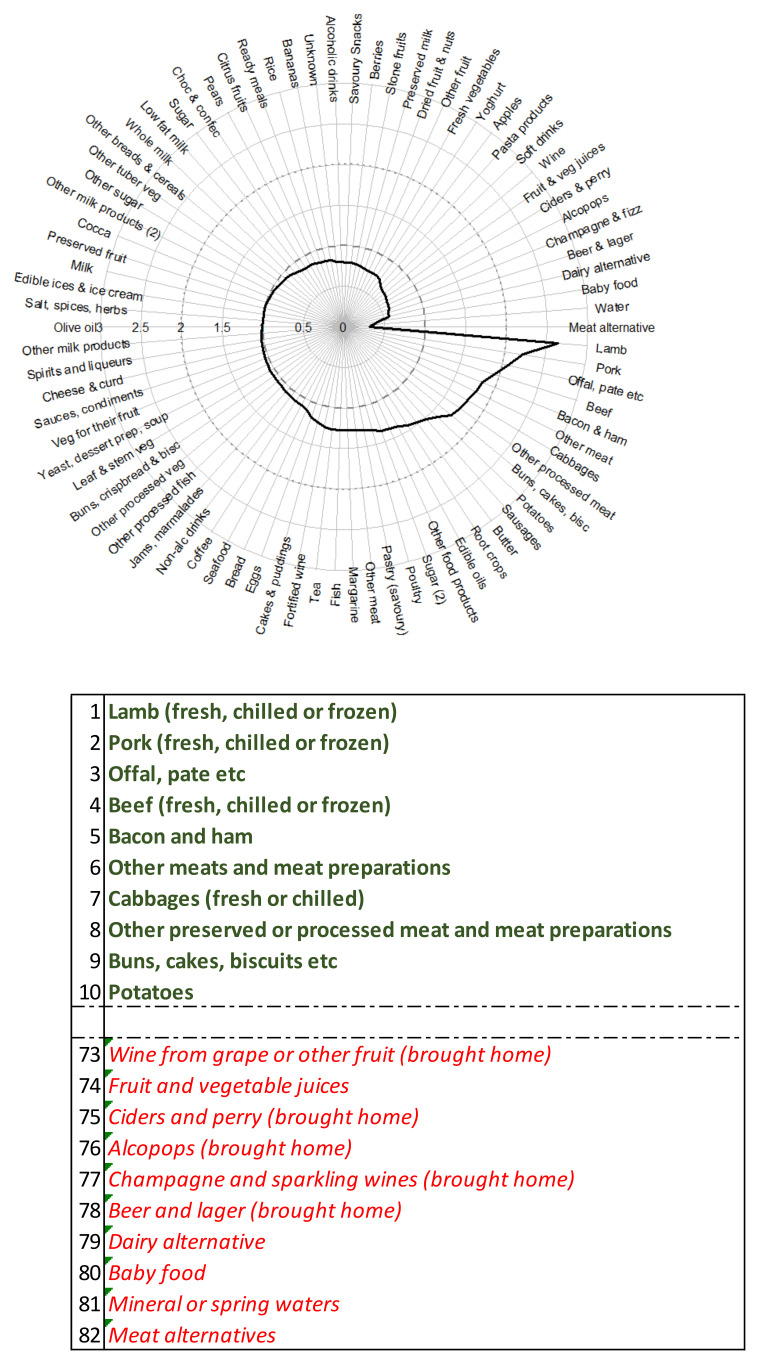
Carnivores (*n* = 49,923).

**Figure A4:**
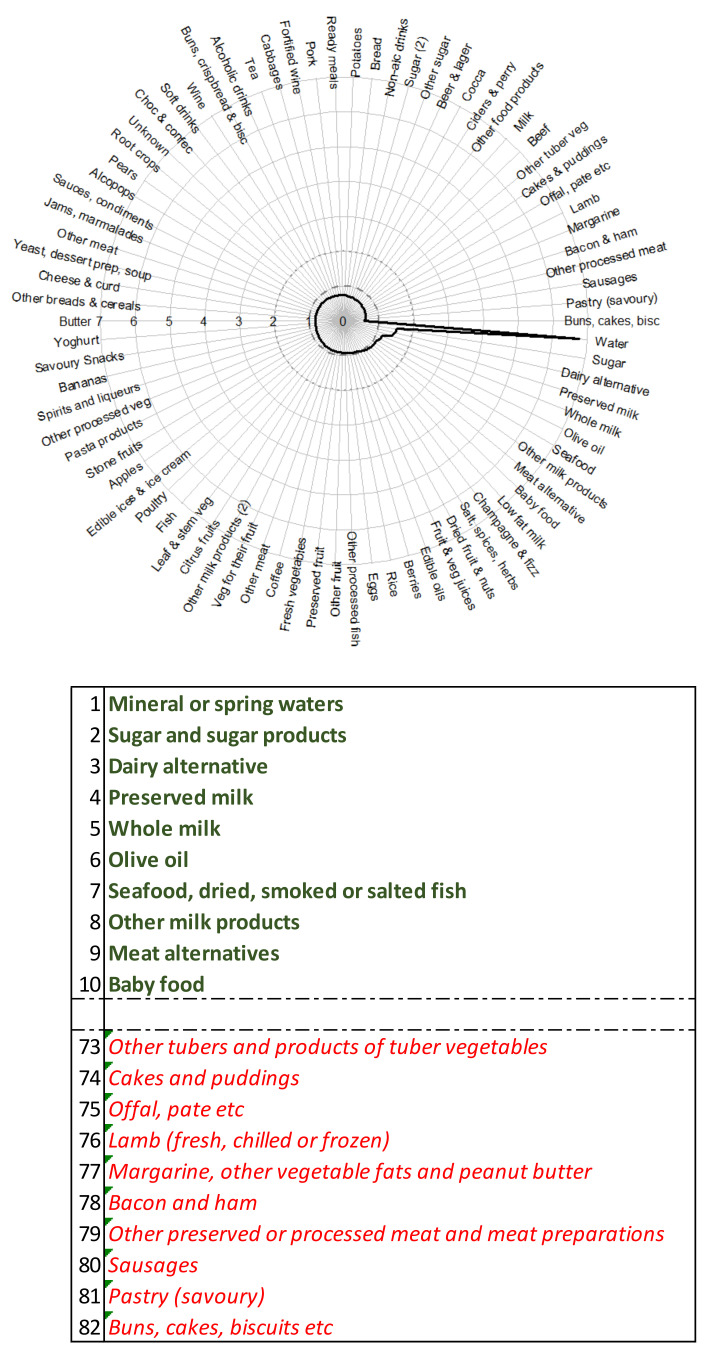
Hydrators (*n* = 22,804).

**Figure A5:**
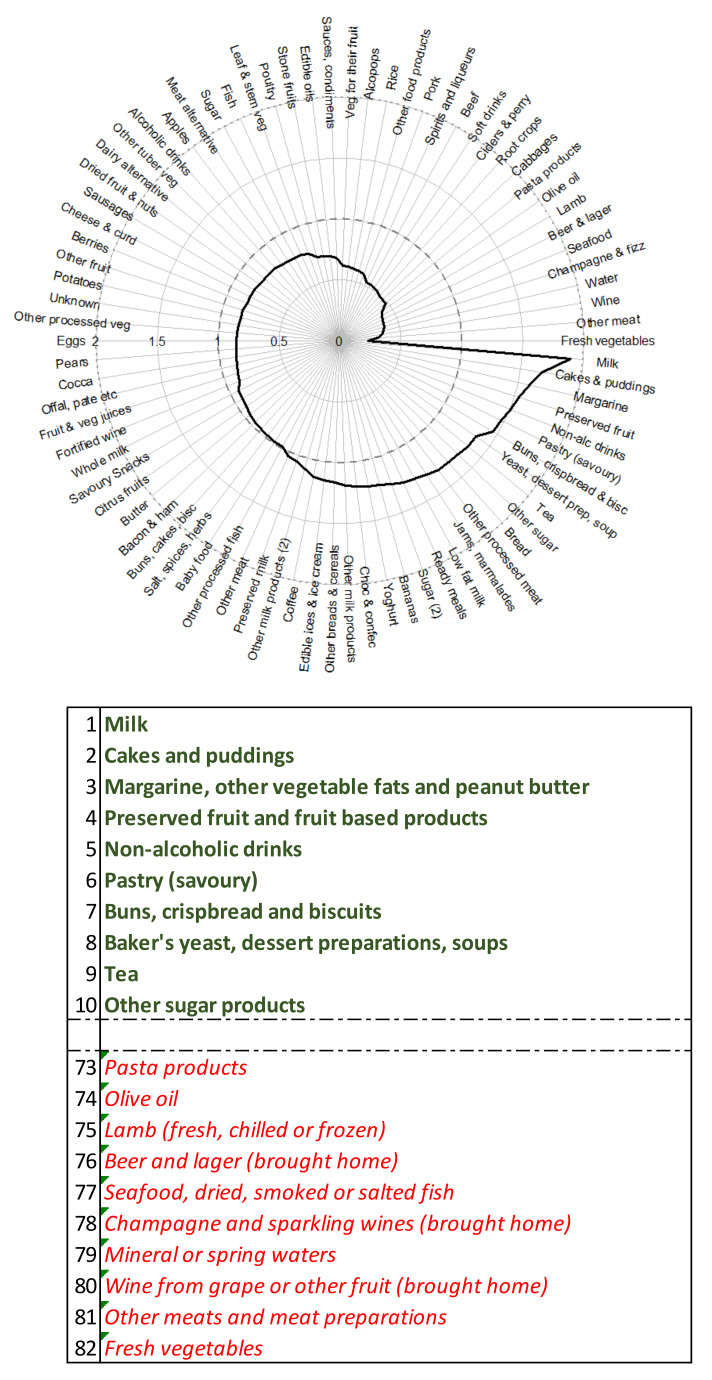
Afternoon tea (*n* = 41,309).

**Figure A6:**
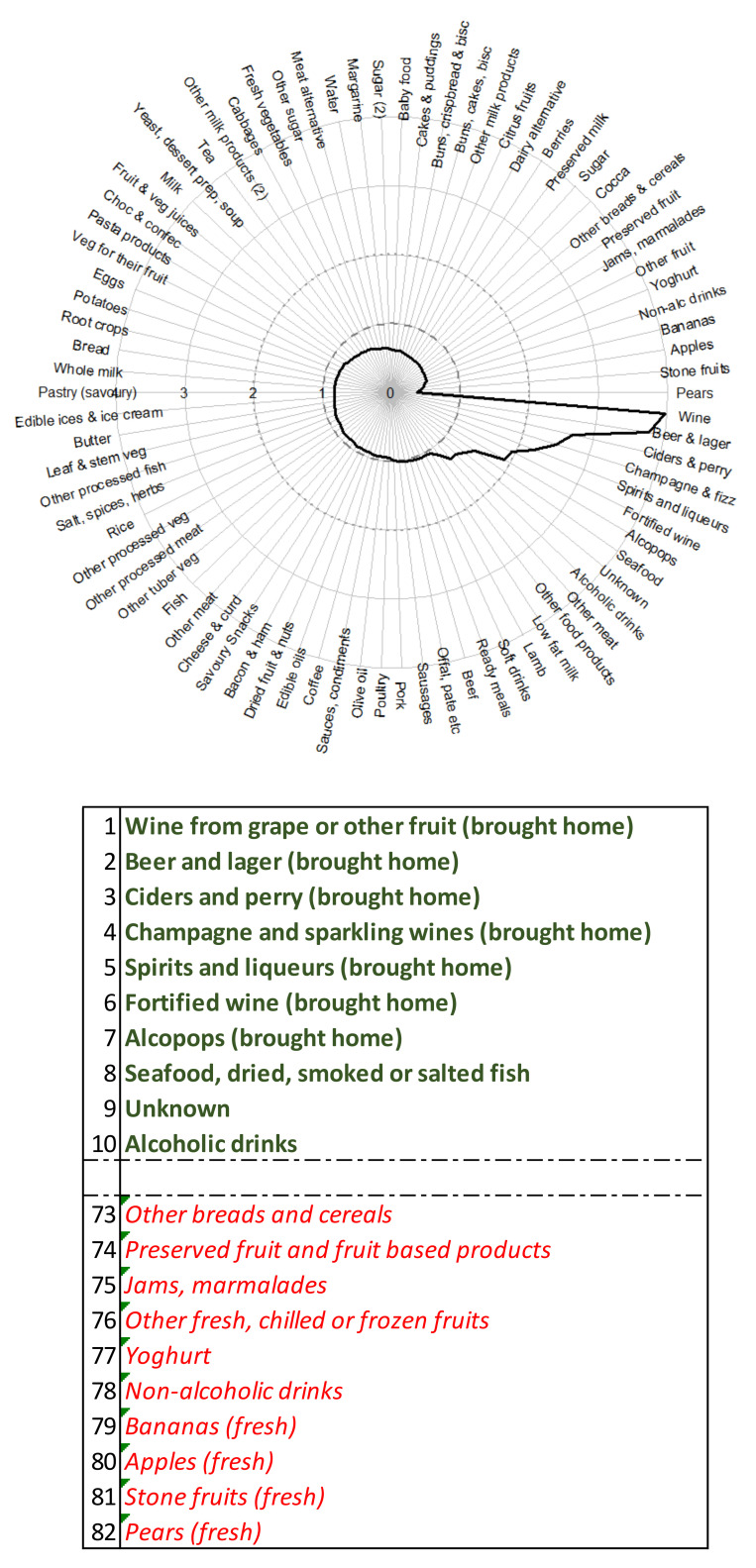
Beer and wine lovers (*n* = 35,486).

**Figure A7:**
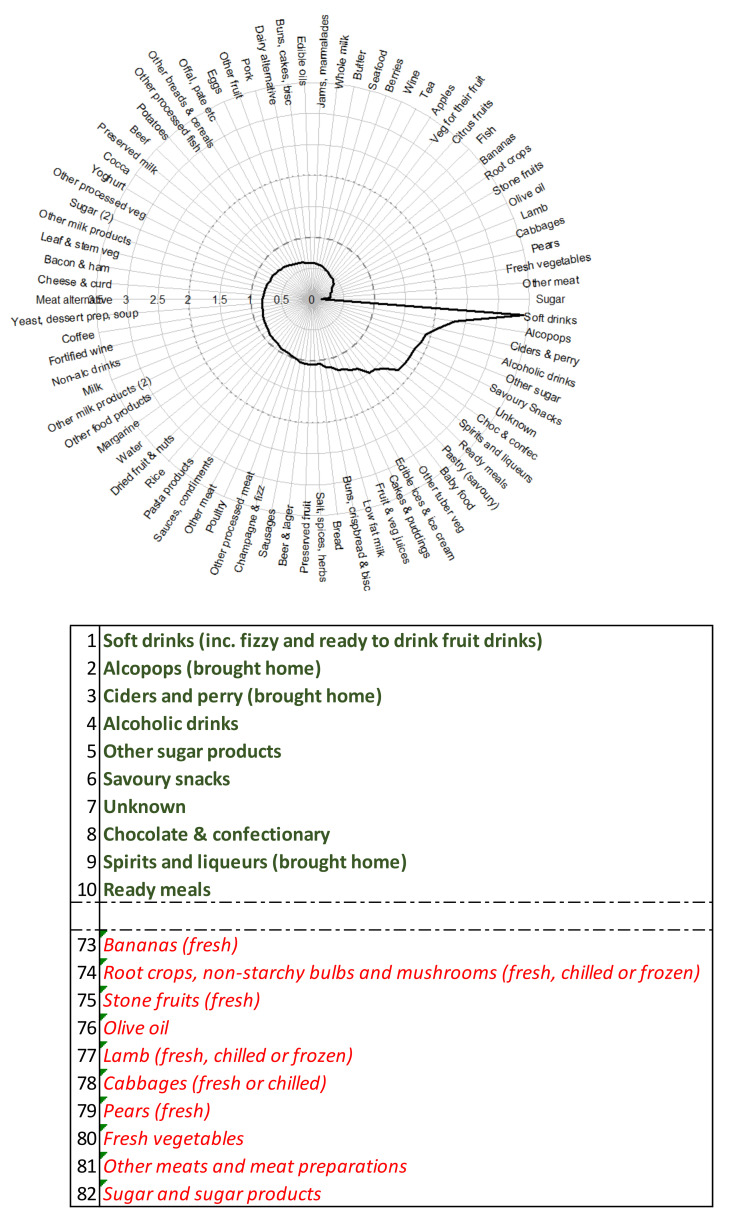
Sweet tooth (*n* = 31,441).

## Data Availability

Due to the commercial nature of the data used in this research, it is not possible for data to be published alongside the manuscript.

## Appendix A. Detailed Information on the Dietary Patterns

### Interpretation of Groupings

Each subsequent page contains the information necessary to profile each loyalty card holder grouping. A loyalty card holder may be purchasing for just themselves, or for a household which may be more than one person. The top of the page contains an ordered radial plot showing how many times MORE or LESS than a typical (average) loyalty card holder in this grouping is purchased in each category of food or drink. For example, a value of 2.0 indicates that they purchase twice as much of this category as a typical customer. A value of 1.0 indicates that they spend exactly the same as a typical household, and a value of 0.5 indicates that they spend half as much. The noteworthy categories are shown around the 3 o’clock position, either categories that are purchased more or items that are purchased less than a typical household.

Below the radial plot are the top and bottom ten categories within this group (i.e., categories in the region of 3 o’clock in the radial plot).
